# The Role of Oxidative Stress and MicroRNAs in Platelet Activation and the Efficacy of Antiplatelet Therapy in Acute Myocardial Infarction

**DOI:** 10.1055/a-2741-4754

**Published:** 2025-11-27

**Authors:** Teodora Vichova, Zuzana Motovska

**Affiliations:** 1 Cardiocenter, Third Faculty of Medicine, Charles University and University Hospital Kralovske Vinohrady, PragueCzech Republic; 2 Department of Pathophysiology, Third Faculty of Medicine, Charles University, PragueCzech Republic

**Keywords:** oxidative stress, miRNA, platelet activation, antiplatelet therapy, ticagrelor, prasugrel

## Abstract

Acute myocardial infarction (AMI) remains a formidable challenge in cardiovascular medicine, necessitating effective antiplatelet therapy to mitigate adverse outcomes. Recent advances have underscored the pivotal role of oxidative stress and micro ribonucleic acids (miRNAs) in regulating platelet activation and modulating the efficacy of antiplatelet agents. This review comprehensively examines the current understanding of how oxidative stress influences platelet function and the regulatory mechanisms of miRNAs in this context. It discusses the dual role of oxidative stress in promoting and impairing platelet activity and its implications for miRNAs as critical modulators of platelet activation, including their potential utility as biomarkers and therapeutic targets. Furthermore, the interaction between oxidative stress, miRNA expression, and antiplatelet drugs is analyzed to elucidate their combined impact on AMI treatment. These insights provide potential pathways to optimize therapeutic strategies, ultimately improving patient outcomes in AMI management.

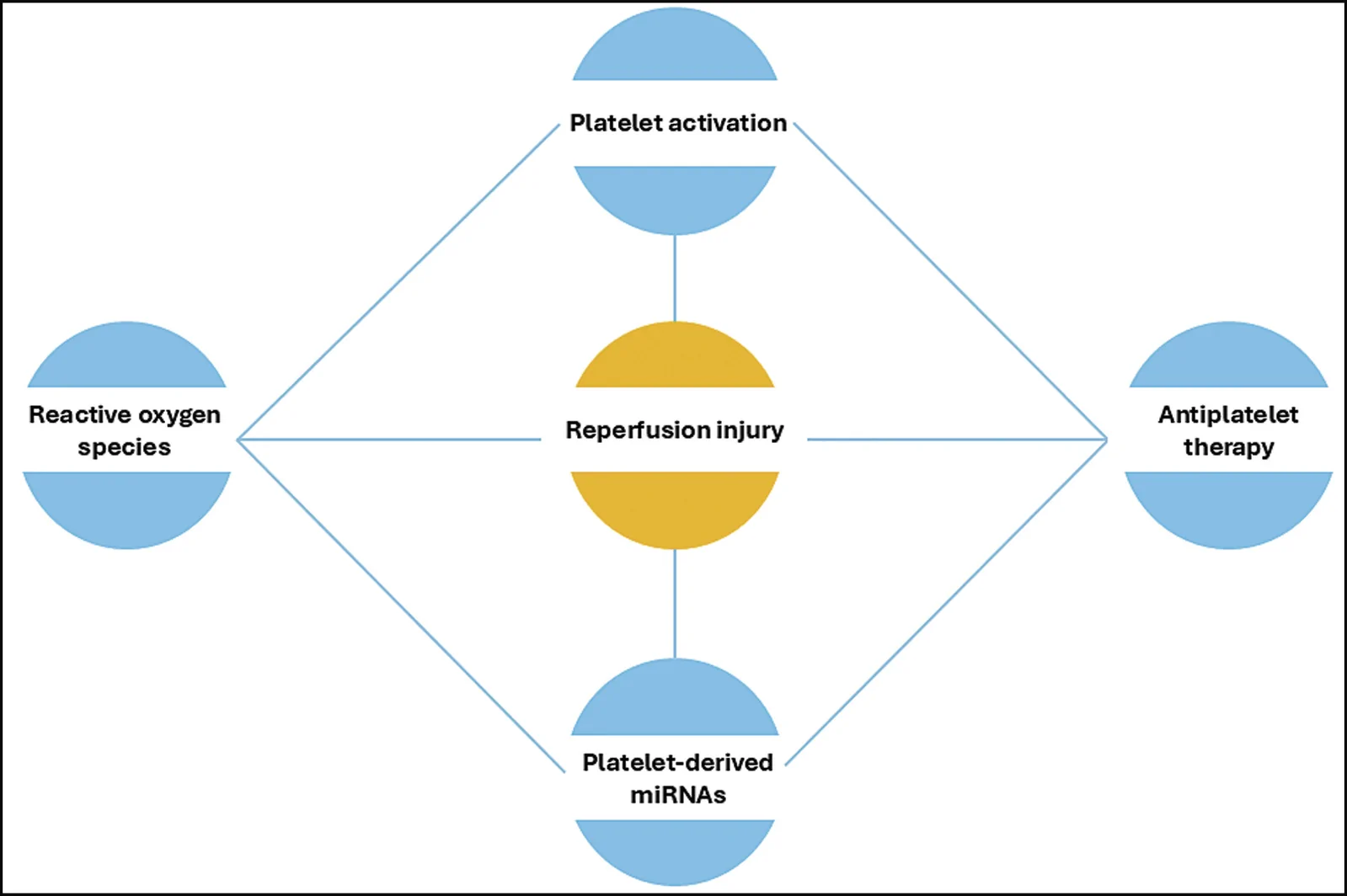

## Introduction

In acute myocardial infarction (AMI), timely reperfusion of the ischemic myocardium improves patient prognosis. However, the abrupt restoration of blood flow and the consequent increase in oxygen levels induce myocardial ischemia–reperfusion injury (IRI), which exacerbates the inflammatory response and elevates the production of reactive oxygen species (ROS). Circulating platelets and leukocytes activate early during reperfusion; their activation is influenced by the duration of the preceding coronary occlusion and correlates with the severity of myocardial injury. 1 Platelets adhere to the damaged endothelium upon reperfusion and infiltrate the ischemic/reperfused (IR) myocardium. By releasing proinflammatory cytokines, chemokines, and ROS, activated platelets further potentiate IRI by forming microthrombi, platelet–leukocyte aggregates, and releasing numerous potent proinflammatory molecules within the affected myocardium. 2 Platelet aggregation and microthrombi formation can occlude microvasculature and impair tissue perfusion despite the restoration of macroscopic blood flow. The expansion of damage in the infarct zone due to IRI can make up to 50% of the final myocardial infarct size. 3 Various imaging studies show that platelets contribute significantly to cardiac reperfusion injury. 1 4 Recent investigations suggest that microRNAs (miRNAs), combined with oxidative stress, may be important modulators of platelet function, potentially influencing outcomes following IRI. 5 6 Nevertheless, a comprehensive overview of the mechanisms responsible for these processes remains absent. This review aims to elaborate on the potential interactions among oxidative stress, platelets, and platelet-derived miRNAs and evaluate their utility as diagnostic tools, predictors of outcome, and treatment targets in managing AMI.

## Platelets and Oxidative Stress

Platelet adhesion and aggregation are central to hemostasis; however, they also contribute to pathological processes such as thrombosis and vessel occlusion, the key events in the development of an acute coronary syndrome (ACS). The oxidative environment in ACS affects platelet function through multiple mechanisms. ROS, produced by ischemic endothelial, vascular smooth muscle cells, cardiomyocytes, and leukocytes, are also generated within activated platelets, making them both producers and targets of oxidative stress. 7 8 ROS are important signaling molecules, promoting megakaryocyte differentiation, and platelet production and activation. 9 At low to moderate levels, ROS enhance platelet adhesion, coagulation, and thrombus formation, increasing thromboembolic risk. However, high ROS levels induce platelet apoptosis, leading to thrombocytopenia and bleeding. 10

ROS can sensitize or directly activate key platelet surface receptors such as glycoprotein IIb/IIIa, and thromboxane receptors, facilitating fibrinogen binding, collagen-induced activation, and platelet aggregation, 9 11 12 and P2Y _12_ and thromboxane receptors. 12 ROS also enhance intracellular calcium mobilization, a critical trigger for platelet activation and degranulation. 13 Additionally, oxidative stress causes glycation, oxidation, and lipid peroxidation of platelet membranes, altering integrity, fluidity, and receptor function, which promotes platelet activation and aggregation. 14 15 These mechanisms contribute to platelet hyperreactivity and thrombus formation in myocardial IRI, leading to vascular occlusion and myocardial injury. ROS-induced platelet activation also plays important roles in several pathological conditions beyond cardiovascular disease—such as cancer-associated thrombosis, 16 inflammation, 17 autoimmune diseases, 18 and neurodegeneration. 19

## Sources of ROS in Platelets

ROS are produced in platelets by several sources, most notably nicotinamide adenine dinucleotide phosphate (NADPH) oxidases (NOX), cyclooxygenase (COX), mitochondrial electron transport chain (ETC), and xanthine oxidase (XO). 20 The primary function of NOX is to catalyze superoxide production from oxygen and NADPH. In human platelets, NOX-1 and NOX-2 are major contributors to production of ROS and key regulators of platelet activation. 21 22 NOX isoforms are inactive at rest and are triggered by agonist stimulation, with NOX-1 predominantly responding to collagen and NOX-2 to thrombin. The dismutation of NOX-2-derived superoxide into hydrogen peroxide is the primary trigger for platelet-mediated thrombus consolidation. Hereditary NOX-2 deficiency reduces platelet recruitment and thrombus growth, highlighting its critical role in platelet activation. 23 NOX-2 also contributes to adverse cardiac remodeling and dysfunction after AMI. 24

ROS activate COX enzymes, primarily COX-1, to produce thromboxane A _2_ (TXA _2_ ) in platelets. COX and peroxidase catalyze the formation of prostaglandin H _2_ , the precursor of TXA _2_ , from arachidonic acid. ROS, including superoxide and hydrogen peroxide, are metabolic byproducts of this process, contributing to the redox environment and influencing platelet function. TXA _2_ is a powerful platelet activator, enhancing aggregation and vascular constriction, exacerbating thrombotic events following AMI. In patients undergoing percutaneous coronary intervention (PCI), platelets demonstrated enhanced TXA _2_ production via ROS-dependent NOX-2. 23

XO catalyzes the oxidation of hypoxanthine to xanthine and subsequently to uric acid, generating hydrogen peroxide and superoxide. Although XO is mainly synthesized in the liver and endothelial cells, platelets can internalize circulating XO or acquire it from endothelial microparticles, enabling localized ROS production. XO-derived ROS enhance platelet activation by modulating redox-sensitive signaling pathways, such as phosphatidylinositol 3-kinase (PI3K)/Akt, mitogen-activated protein kinase (MAPK), and protein kinase C (PKC). Elevated XO activity is seen in AMI, and it has been implicated in myocardial stunning after ischemia. 25 26 Platelets contain functional mitochondria essential for adenosine triphosphate (ATP) production through oxidative phosphorylation and are significant sources of ROS. During mitochondrial respiration, ROS are byproducts of electron transport chain (ETC) activity, involving complexes I to V. Electrons supplied by nicotinamide adenine dinucleotide (NADH) and flavin adenine dinucleotide (FADH _2_ ) are transferred through the ETC, reducing oxygen to water at complex IV. Throughout this process, electron leakage predominantly occurs at complex I and complex III. Leaked electrons reduce molecular oxygen to form superoxide anion. 27 Elevated ROS levels can impair mitochondrial membrane potential, promoting further ROS generation in a positive feedback loop that can lead to mitochondrial dysfunction, platelet hyperreactivity, or apoptosis.

## Modulation of Platelet Activity by ROS via Apoptosis and Ferroptosis

### Apoptosis

Apoptosis is a regulated process leading to cell self-destruction. While traditional apoptosis involves nuclear DNA fragmentation, platelet apoptosis shares many morphological and biochemical features, primarily driven by mitochondrial signals. 28 ROS play a central role in platelet homeostasis: at low to moderate levels, they promote activation, while at high levels, they trigger apoptosis and ferroptosis. Platelets contain both pro- and anti-apoptotic proteins. During apoptosis, survival signals are suppressed and the pro-apoptotic signals trigger mitochondrial depolarization, cytochrome C release, and caspase cascade activation. Caspase-3 then drives phosphatidylserine (PS) externalization, promoting both clearance by phagocytes and a procoagulant platelet phenotype that enhances thrombin generation and clot stability. 29 30 Excessive platelet apoptosis may cause thrombocytopenia, and mitochondrial apoptosis further amplifies ROS production in a feedback loop. 10 31 Platelets may also undergo apoptosis via extrinsic pathways triggered by cytokines like tumor necrosis factor-α (TNF-α), linking inflammation to thrombosis, although this pathway in platelets remains underexplored.

### Ferroptosis

Ferroptosis is a form of iron-dependent cell death, driven by ROS-mediated lipid peroxidation, and mechanistically differs from apoptosis and necrosis. 32 It occurs under oxidative stress when glutathione peroxidase (GPx) 4, a key enzyme against lipid peroxidation, is depleted. 33 This disrupts iron homeostasis, leading to increased cytosolic iron from ferritin and mitochondria, and the release of hemin from hemoglobin. Hemin activates heme oxygenase-1 (HO-1), producing ferrous iron, biliverdin, and carbon monoxide, further contributing to iron overload and platelet ferroptosis. 34 35 Both ferroptosis and apoptosis contribute to platelet dysfunction and cardiomyocyte injury during AMI. Platelets exacerbate cardiomyocyte damage by reducing protective lipid species like stearic acid–phosphatidylcholine. 36 Melatonin, an antioxidant, inhibits hemin-induced ferroptosis in platelets, suggesting its potential in treating thrombotic and thrombocytopenic conditions. 35

## Platelet Antioxidant Systems

### Endogenous Antioxidant Systems

Antioxidants maintain platelet redox homeostasis by neutralizing ROS and modulating redox-sensitive signaling. Platelet defenses comprise enzymatic antioxidants such as catalase (CT), superoxide dismutase (SOD), GPx, HO-1, and glutathione-S-transferase (GST), or thioredoxin (TRX), along with non-enzymatic molecules including glutathione (GSH). 37 Since platelet activation generates ROS that amplify further activation, antioxidants help limit cytotoxic effects and promote antithrombotic actions by scavenging ROS and enhancing nitric oxide (NO) bioavailability.

### Exogenous Antioxidant Systems

Vascular antioxidant systems, similar to platelet mechanisms, include enzymes such as SOD, CT, GPx, GST, and HO-1, along with extracellular antioxidants like the GSH/GSSG pair and GPx3. 38 These systems help suppress ROS and reactive nitrogen species (RNS)-driven oxidative reactions that promote platelet activation, thus contributing to redox homeostasis. Plasma antioxidants, such as SOD and CT, neutralize ROS near the platelet membrane, preventing oxidative damage to membrane lipids and proteins. Other antioxidants, such as TRX and vitamin C, are actively transported into platelets via specific membrane transporters, while NO, a key antiplatelet and vasodilatory molecule, diffuses freely into platelets, modulating ROS levels to suppress activation and aggregation. The summary of platelet antioxidant systems is in Supplementary Table S1 (available in the online version only).

## Protective Roles of Platelets in Myocardial Reperfusion Injury

Although platelets are traditionally seen as contributors to myocardial IRI through their prothrombotic and proinflammatory roles, emerging evidence suggests they also have protective effects during reperfusion. These include the release of cardioprotective molecules, mitophagy, and interactions with inflammatory cells and the endothelium.

### Release of Cardioprotective Molecules

Platelets release bioactive molecules such as adenine nucleotides, TXA _2_ , serotonin, and transforming growth factor-β1 (TGF-β1), which stimulate endothelial NO production, promoting cardioprotection. 39 40 TGF-β1, released during platelet activation, modulates inflammation and supports cardiac repair, its reduced expression being linked to higher mortality and AMI incidence. 41 Additionally, sphingosine-1-phosphate, released in the course of platelet activation and thrombus development, provides further cardioprotection. 42 Platelet-released stromal cell-derived factor-1α (CXCL12) promotes progenitor cell migration for tissue repair and enhances cardiac function after AMI by delaying cardiomyocyte death. 43

### Platelet Mitochondrial Uncoupling

Platelets rely on both glycolysis (60%) and oxidative phosphorylation (40%) for ATP production. 44 Mitochondrial function is crucial for energy, calcium homeostasis, and ROS signaling, all of which influence platelet activation and thrombus formation. 45 Mitochondrial uncoupling, a state when protons leak back into the mitochondrial matrix without generating ATP, reduces membrane potential and ROS production. 46 Although initially thought to indicate dysfunction, endogenous uncoupling proteins suggest it regulates several biological processes. 47 48 Mild uncoupling can decrease platelet activation by limiting ATP and ROS production, 49 while excessive uncoupling leads to metabolic stress, ATP depletion, and compensatory glycolysis, which indirectly affects ROS levels. Mitochondrial uncoupling may be a potential target for modulating platelet function and decreasing thrombotic risk. 50

### Mitophagy

Mitophagy, a selective degradation pathway, clears damaged mitochondria to maintain mitochondrial quality under hypoxic stress. 51 During early stages of cardiac IRI, it removes dysfunctional mitochondria while preserving functional ones to support platelet activation. Later, sustained hypoxia triggers more extensive mitophagy, reducing mitochondrial content and platelet activation. This mechanism limits excessive platelet activation and reduces myocardial injury via negative feedback. 52

### Interactions with Leukocytes and Endothelium, Modulation of Inflammation

Although these interactions can promote injury, platelets may facilitate reparative leukocyte responses and vascular repair under controlled activation. Some platelet-derived extracellular vesicles (EVs) and microparticles have anti-inflammatory effects, which may help limit neutrophil infiltration and reduce endothelial damage. 53 The contribution of platelets to cardioprotection is not yet fully understood. In most pathological contexts, platelet overactivation exacerbates injury. Consequently, therapeutic strategies typically aim to inhibit platelet activity.

## Platelet miRNAs

miRNAs are small non-coding RNAs that modulate gene expression by interacting with target mRNAs after transcription. They influence up to two-thirds of the human genome and are involved in numerous cellular processes. 54 Although platelets contain fewer miRNAs compared with nucleated cells, they carry approximately 30% of mature human miRNAs. Up to 70% of the extracellular miRNAs, including that circulating in the blood, are platelet-derived. 55 Circulating miRNAs are stable and act as paracrine mediators between platelets, inflammatory cells, endothelium, and cardiomyocytes. Circulating miRNAs can be transported: (a) within microvesicles or extracellular vesicles (EV) shed from plasma membrane; (b) membrane-bound small vesicles of endosomal origin—exosomes; (c) in complexes with Argonaute 2 protein; (d) carried by high-density lipoprotein, or to the RNA-binding protein nucleophosmin; or (e) released from cells in apoptotic bodies. Platelet-derived exosomes can be taken up by recipient cells including leukocytes or endothelial cells, participating in inflammation, thrombosis, and atherogenesis. 55 Platelets retain megakaryocyte-derived mRNAs, as well as possess the translational machinery (e.g., rough endoplasmic reticulum, polyribosomes), enabling de novo protein synthesis. 56 miRNAs regulate key platelet functions—including activation, aggregation, secretion, and adhesion—by modulating the translation of key proteins such as integrin/cytoskeletal receptors, platelet secretion machinery (e.g., VAMP8), and signaling intermediates. 5 6 57 Interestingly, it has been observed that during activation, nearly half of the more than 700 quantified platelet proteins were modulated, while the platelet transcriptome remained largely unchanged owing mostly to the alterations in miRNA expression. 58

## Platelet miRNAs and Oxidative Stress Interactions

Growing evidence highlights the dual role of miRNAs as both regulators and targets of oxidative stress-related pathways. 59 miRNAs can promote or inhibit ROS production by modulating transcription factors and genes involved in ROS generation and scavenging. 60 Similarly, ROS alter miRNA expression and activity at multiple levels. The complex interactions impact key processes involved in IRI such as platelet function, inflammation, angiogenesis, fibrosis, and ventricular remodeling. 61 62

### Impact of ROS on miRNAs

#### Regulation of miRNAs Production at Transcriptional Level

miRNA expression is influenced by various stressors, including oxidative stress, which acts through transcription factors like nuclear factor kappa B (NF-κB) and nuclear factor E2-related factor 2 (Nrf2). In megakaryocytes, these changes shape the miRNA content of newly formed platelets. 59 63 The regulation of miRNA transcription occurs not only in megakaryocytes but also in platelets. Although anucleate, platelets retain a functional transcriptional and posttranscriptional regulatory system including pathways typically found in nucleated cells, such as NF-κB, sirtuin 1 (SIRT), and Nrf2. 64 65 NF-κB generally promotes a pro-thrombotic, inflammatory phenotype in response to oxidative stress, while SIRT and Nrf2 pathways exert protective effects by attenuating ROS-mediated damage.

For the overview of selected miRNAs and their impact on signaling pathways, see Table 1 .

**Table 1:** Platelet miRNA overview—their relation to oxidative stress and potential as biomarkers

miRNA	Signaling pathway	Effect on tissues	Use as biomarker/predictor
**miRNA-223**	Blocks the NF-κB and MAPK pathways, targets the NLRP3 and ICAM-1 gene and inhibits its expression 55 125	Protects cardiomyocytes from oxidative stress-induced apoptosis, promotes proliferation, migration, induces fibrosis, inhibits endothelial inflammation 55 125	Negatively correlated with risk of incident AMI 86 ↑ levels predicted CVD death in CAD patients 89 Associated with higher risk of ischemic endpoint within 30 days and 1 year after AMI 120
**miRNA-126**	Activates ERK1/2 signaling pathway, SIRT1/Nrf2 signaling pathway 74	Inhibits oxidative stress and reperfusion injury, promotes angiogenesis in MI patients 126 ; decrease in the levels of pro-inflammatory cytokines	Positively correlated with risk of incident AMI ↑ Levels predicted ↑ risk of MACE after PCI 126 Predictor of thrombotic events after AMI 120
**miRNA-21**	Deficiency activates NF-κB pathway, regulates TGF-β/Smad7 signaling 84 127	↓ Apoptosis, reduces inflammation, preserves cardiac function, and limits adverse remodeling after AMI; continuous expression may lead to cardiac hypertrophy and obesity 127 128	↑ Levels in patients with ACS, earlier than troponins 129 Strong predictive ability for MACE at 3 months following AMI 130
**miRNA-24**	Inhibits NF-κB/TNF-α pathway, activates Keap1/Nrf2, activates the Nrf2/heme oxygenase-1 signaling pathway 85 131	Inhibition of oxidative stress, reduction of apoptosis in MI reperfusion, attenuates fibrosis following AMI, blocking increased angiogenesis and ↓ apoptosis post-AMI 85 132 133	↓ Levels in AMI, correlated with troponin 119
**miRNA-197**	Inhibits the TLR4/NF-κB/NLRP3 pathway 134	Mitigates inflammation and endothelial dysfunction 134	↑ Levels predicted CVD death in CAD patients 89 Negatively correlated with risk of incident AMI 86
**miRNA-96**	Suppresses caspase 1 expression, negatively targets IGF1R, Bax protein 135 136	Inhibits pyroptosis in ischemia-reperfusion injury, ↓ endothelial inflammation, migration and tubule formation, suppresses apoptosis, regulates DNA repair, carcinogenic effect 136	↑ Levels in AMI mice 137
**miRNA-150**	Activates PI3K/Akt/eNOS pathway, ↓ expression of cleaved caspase 3 and Bax, ↑ expression of Bcl-2 138	↓ Apoptosis, reduces inflammation; ↑ ischemia-induced neovascularization, ↓ associated with maladaptive remodeling 138 139 140	↑ Levels in AMI 141 142 ↓ Levels associated with advanced heart failure, its severity and outcome 140
**miRNA-191**	Represses OXSR1 + vs MAPK/NF-κB signaling pathway 143	Protection from apoptosis; inhibits angiogenesis 143 144	↓ Levels in AMI 145

Abbreviations: AMI, acute myocardial infarction; AP1, activator protein 1; Bax, bcl-2-like protein; CAD, coronary artery disease; CVD, cardiovascular disease; EBP, emopamil binding protein; ERK1/2, extracellular signal-regulated kinase ½; ICAM-1, intercellular adhesion molecule 1; IGF1R, insulin-like growth factor 1 receptor; Keap1, Kelch-like ECH-associated protein 1; MACE, major adverse cardiovascular events; MAPK, mitogen-activated protein kinase; miRNA, micro ribonucleic acid; NF-κB, nuclear factor kappa B; NLRP3, NOD-like receptor protein 3; OXSR1, oxidative stress responsive kinase 1; PCI, percutaneous coronary intervention; PI3K/Akt/eNOS, phosphoinositide 3-kinase/protein kinase B/ endothelial nitric oxide synthase; SIRT1/Nrf2, sirtuin 1/nuclear factor E2-related factor 2; TGF-β/Smad7, transforming growth factor β.

#### Regulation of miRNAs at Post-transcriptional Level

At the post-transcriptional level, cellular stress affects miRNA maturation. miRNAs are generated as primary transcripts (pri-miRNAs), converted into pre-miRNAs by the nuclear RNase III enzyme Drosha and its cofactor DGCR8, and then transported to the cytoplasm via exportin-5. In the cytoplasm, Dicer1, a member of the RNase family, shortens pre-miRNAs into 22-nucleotide duplexes, and one functional strand is assembled with Argonaute proteins (Ago1–Ago4) into the miRNA-induced silencing complex (miRISC), enabling miRNA to exert its post-transcriptional regulatory function. 66

(a) ROS can disrupt miRNA processing by RNases such as DROSHA, DGCR8, or Dicer1, enhancing or suppressing miRNA maturation. 67 68
(b) Stress can impair pre-miRNA export from the nucleus, such as when exportin-5 becomes saturated, reducing mature miRNA levels. 63
(c) Modifications of Ago2 proteins reduces its binding to target mRNA, leading to deficiencies in miRNA function. 69 Platelet-specific Ago2 deletion in mice increases platelet volume, clot retraction, and integrin expression. 70

### Impact of miRNAs on ROS Production and Clearance

Platelet miRNAs can reciprocally regulate transcriptional and signaling pathways, forming a feedback loop that impacts oxidative stress. 71 For example, miRNA-25 and miRNA-146a regulate NOX enzymes and mitochondrial ROS production. 72 Upregulation of miRNA-24 promotes endothelial regeneration following balloon injury by reducing oxidative stress via the Nrf2/HO-1 pathway and modulating antioxidant enzymes like SOD and GPx. 73 Overexpression of miRNA-126 decreases ROS and enhances SOD and GPx activity. 74 These mechanisms balance platelet activation and inhibition under oxidative conditions.

## miRNAs and Their Roles in Regulation of Platelets and Other Cells

miRNAs are transferred bidirectionally between platelets and their environment. 53 75 Under oxidative stress, activated endothelial cells, leukocytes, and megakaryocytes release extracellular vesicles containing miRNAs, which platelets can internalize. The internalization modulates signal-dependent translation processes and platelet function. Many circulating microRNA levels change with antiplatelet therapy, reflecting their role in platelet function.

miRNA-223 is one of the most prevalent and well-characterized miRNAs in platelets. It modulates platelet activation by targeting genes associated with inflammatory responses and integrin pathways, including the P2Y _12_ receptor and NF-κB pathway. 76 miRNA-223 influences platelet–leukocyte interactions via EV transfer and plays a significant role in macrophage function during inflammatory stimulation and atherosclerosis. 77 Additionally, miRNA-223 regulates intercellular adhesion molecule 1 (ICAM-1) expression, decreasing inflammation following thrombosis. 55 On the other hand, platelet miRNA-223 promotes myocardial IRI and ferroptosis in cardiomyocytes following AMI by affecting lipid content in cardiomyocyte membranes. 36

miRNA-126 is one of the most highly expressed miRNAs in platelets and is closely associated with platelet activation markers like P-selectin. 78 Platelets serve as a primary contributor to circulating miRNA-126, which promotes thrombus formation. 79 In cardiomyocytes, miRNA-126 inhibits oxidative stress, inflammation, macrophage recruitment and fibrosis. 80

miRNA-21, produced in platelets, is involved in protecting against myocardial IRI by suppressing apoptosis and cardiac remodeling. 81 82 Inhibition of miRNA-21 lowers circulating platelet counts and decreases platelet release of TGF-β1. 83 Overexpression of miRNA-21 reduces hydrogen peroxide-induced ROS activity and cardiomyocyte apoptosis, playing a key role in ROS-mediated injury. 84

Platelets and the vascular endothelium exhibit substantial expression of miRNA-24, with dysregulation linked to endothelial dysfunction and cardiovascular diseases. During IRI in mice hearts, miRNA-24–3p expression decreases, but miRNA mimics reduce cardiomyocyte apoptosis caused by ROS. 85 Blocking endothelial miRNA-24 reduces infarct size by inhibiting endothelial cell death and promoting vascular growth. 85

miRNA-197 is highly expressed in platelets. 86 Although its function in platelet activation remains unclear, it might play a part in lipid abnormalities associated with metabolic syndrome, promoting cardiovascular disease (CVD) progression. 87 Lower blood levels of miRNA-197 are observed in individuals with type 2 diabetes, suggesting it could be a prognostic biomarker for future CVDs. 88 89

miRNA-96 regulates vesicle-associated membrane protein 8 (VAMP8)/endobrevin, a protein involved in platelet degranulation. The expression of VAMP8 differs in hyperreactive versus hyporeactive platelets. When switching from clopidogrel to ticagrelor, miRNA-96 levels increased. 57

An overview of selected miRNAs, their relation to oxidative stress, and their potential as biomarkers is summarized in Table 1 , and impact of antiplatelet therapy on miRNA levels in Table 2 . Interactions of platelet miRNAs and oxidative stress are depicted in Fig. 1 .

**Table 2:** miRNAs and their relation to antiplatelet therapy and platelet function

miRNA	Dynamics on antithrombotic agents	Thrombocyte receptor miRNA	Impact on platelet function/correlation with other platelet reactivity assessment methods
**miRNA-223**	↓ On ticagrelor vs. clopidogrel 105 106 ↓ On prasugrel and prasugrel + ASA 5 104 ↓ On ASA + clopidogrel in HTPR vs. LTPR 155 ↑ On ticagrelor vs. prasugrel 105 107 and ticagrelor vs. clopidogrel 107	PAR1/PAR4, P2Y _1_ / P2Y _12_ , SMOC1, Factor XIII	Positive correlation with platelet function (ADP-induced PAG) 105 ↑ Platelet inhibition on dose escalation of ASA + prasugrel, assessed by plate aggregometry and thromboxane B _2_ + VerifyNow 5 ↓ Levels correlated with PRI (VASP) on ASA + clopidogrel, determined low responders 156 Correlated with ADP-stimulated P-selectin expression circulating levels 104 ↑ In high responders on clopidogrel compared with low responders (PAG by LTA) 156
**miRNA-126**	↑ On ticagrelor vs. prasugrel 107 ↓ On ASA 78 ↓ On ticagrelor vs. clopidogrel 105 106 ↓ Prasugrel and prasugrel + ASA 5 ↓ On ASA + clopidogrel in HTPR vs. LTPR 155	PAR1/PAR4, P2Y _1_ / P2Y _12_ , PLXNB2, ADAM9	Correlates with P-selectin expression 78 ↓ Levels on ticagrelor increasing platelet inhibition on dose escalation of ASA + prasugrel assessed by plate aggregometry and thromboxane B _2_ + VerifyNow 5 Attenuated platelet activity via downregulation of disintegrin and metalloproteinase-9 (ADAM9) 157
**miRNA-21**	↓ On ticagrelor vs. clopidogrel 105 ↑ On ticagrelor compared with prasugrel 105 107 and ticagrelor vs. clopidogrel 107	PAR1/PAR4, P2Y _1_ / P2Y _12_ , WASp	Positive correlation with platelet function (ADP-induced PAG) 105 ↑ In high responders on clopidogrel compared with low responders (PAG by LTA) 158 Correlated with ADP-stimulated P-selectin expression circulating levels 104
**miRNA-24**	↓ On prasugrel vs. ASA 104 ↑ ASA + clopidogrel in clopidogrel resistance 159	PAR1/PAR4, P2Y _1_ / P2Y _12_	Correlated with ADP-stimulated P-selectin expression circulating levels 104 Inversely correlated with inhibition of platelet aggregation (VerifyNow) 159
**miRNA-197**	↓ On ticagrelor vs. clopidogrel 105 ↓ On prasugrel vs. ASA 104	PAR1/PAR4, P2Y _1_ / P2Y _12_	No correlation with ADP-stimulated P-selectin expression circulating levels 104
**miRNA-96**	↑ On ASA + ticagrelor vs. ASA + clopidogrel 106	VAMP8/endobrevin	↑ Levels on ticagrelor compared with clopidogrel 106
**miRNA-150**	↓ On ticagrelor compared with clopidogrel 106 ↓ Prasugrel and prasugrel + ASA 5 ↑ On ticagrelor vs. prasugrel and clopidogrel 107 ↑ Clopidogrel + ASA in HTPR vs. LTPR 155	NA	Increasing platelet inhibition on dose escalation of ASA + prasugrel assessed by plate aggregometry and thromboxane B _2_ additionally assessed by VerifyNow 5
**miRNA-191**	↓ Prasugrel and prasugrel + ASA 5 104	NA	Increasing platelet inhibition on dose escalation of ASA + prasugrel assessed by PAG and thromboxane B _2_ additionally assessed by VerifyNow 5 Correlated with ADP-stimulated P-selectin expression

Abbreviations: ADAM9, A disintegrin and a metalloprotease 9; ADP, adenosine diphosphate; ASA, acetylsalicylic acid; HTPR, high on-treatment platelet reactivity; LTA, light transmission aggregometry; LTPR, low on-treatment platelet reactivity; miRNA, micro ribonucleic acid; PAG, platelet aggregometry; PAR, protease-activated receptor; PLXNB2, plexin B2; SMOC1, secreted modular calcium-binding protein 1; VAMP8, vesicle-associated membrane protein 8; WASp, Wiskott-Aldrich syndrome protein.

**Fig. 1:**
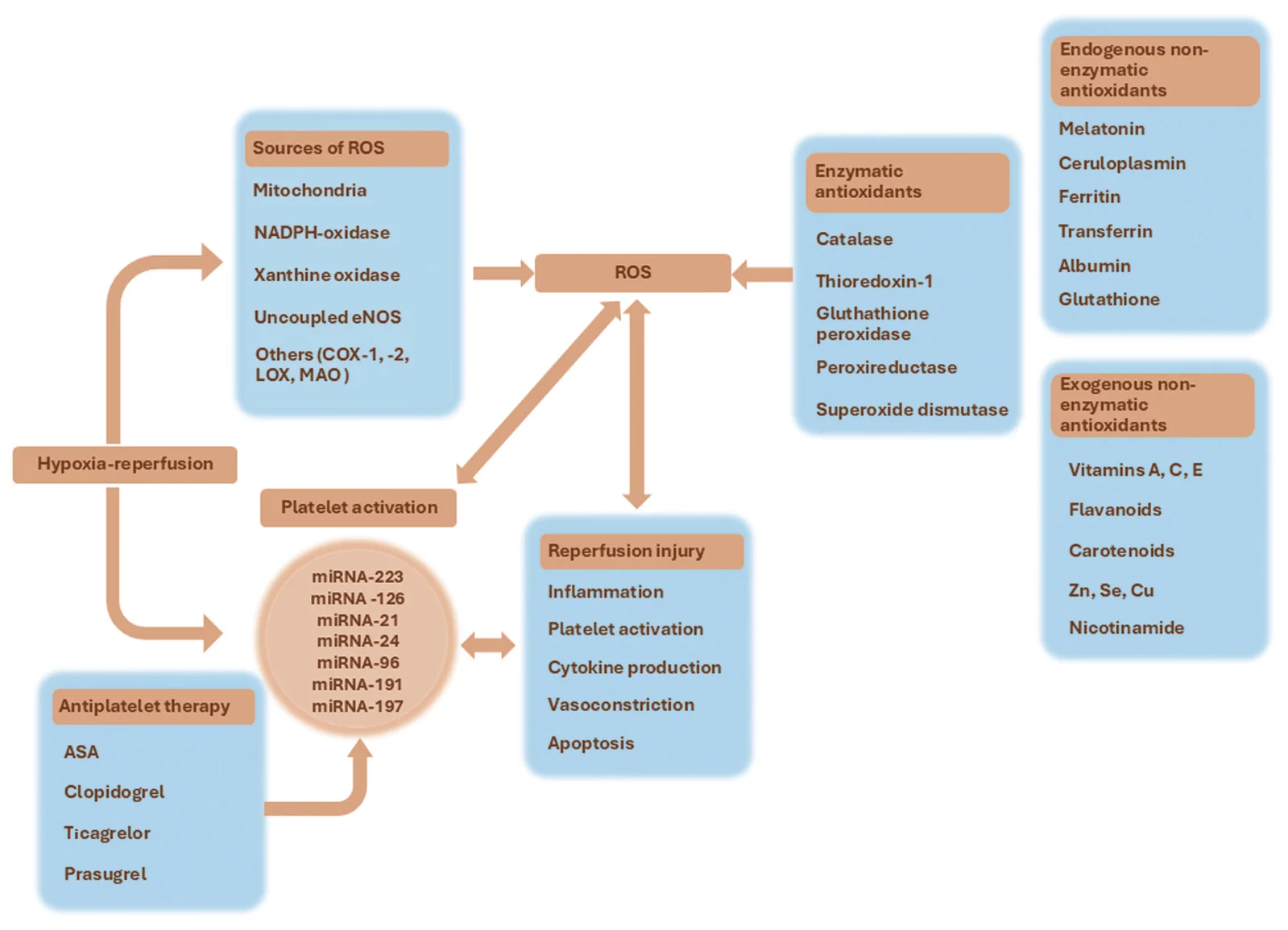
Interplay between oxidative stress, platelets, and platelet-derived miRNAs. ASA, acetylsalicylic acid; COX, cyclooxygenase; Cu, copper; LOX, lipoxygenase; MAO, monoamine oxidase; miRNA, micro ribonucleic acid; NADP, nicotinamide adenine dinucleotide phosphate oxidase; ROS, reactive oxygen species; Se, selenium; Zn, zinc.

## Antiplatelet Therapy, Oxidative Stress, and miRNAs

Following an ACS, a combination of oral aspirin (acetylsalicylic acid [ASA]) and P2Y _12_ receptor antagonists represents the mainstay of antiplatelet therapy.

### General Modes of Action of Antiplatelet Agents

Aspirin irreversibly inhibits platelet COX-1, blocking TXA _2_ synthesis via the arachidonic acid pathway. However, since it inhibits only a single pathway of platelet activation, aspirin alone is less effective for preventing thrombotic events following AMI compared with combination therapy. 90 Platelet activation involves multiple signaling pathways, particularly in the prothrombotic environment following AMI.

The P2Y _12_ receptor is pivotal for platelet aggregation by regulating adenosine diphosphate (ADP)-driven granule release and inhibiting prostacyclin signaling. Clopidogrel, a widely used P2Y _12_ antagonist in ACS and PCI, has limitations due to delayed activation, drug interactions, and metabolic variability due to undergoing metabolization via cytochrome (CYP) enzymes. 91 Newer P2Y _12_ inhibitors—prasugrel, ticagrelor, and cangrelor—offer faster, more reliable inhibition. Prasugrel, like clopidogrel, is a CYP-activated prodrug and acts irreversibly. In contrast, ticagrelor and cangrelor are direct-acting, reversible inhibitors with rapid onset. Overall, prasugrel and ticagrelor show greater potency and consistency than clopidogrel. 92 93

### Impact of Oxidative Stress on Antiplatelet Agents' Efficacy

Oxidative stress increases platelet reactivity by enhancing surface receptors activation (e.g., GPIIb/IIIa, P2Y _12_ ) and promoting signaling pathways leading to aggregation. This hyperactivity reduces antiplatelet efficacy. ROS can bypass aspirin's action through COX-independent activation via isoprostanes, 14 94 95 or interfere with CYP activity, 96 reducing P2Y _12_ inhibitor activation. Oxidative stress is linked to high on-treatment platelet reactivity (HTPR) in individuals receiving clopidogrel. 97 In populations with elevated oxidative stress, such as diabetes, chronic kidney disease, or AMI, increased aspirin resistance, clopidogrel non-responsiveness, and higher thrombotic risk were observed despite receiving DAPT. 97 98

### Impact of Antiplatelet Therapy on Oxidative Stress

#### Aspirin

There are emerging data regarding the antioxidant and anti-inflammatory properties of antiplatelet therapy. Aspirin exerts anti-inflammatory effects by inhibiting prostaglandin synthesis, a process that also produces ROS as metabolic byproducts. 99 Furthermore, aspirin induces the expression of HO-1, an enzyme with cytoprotective and antioxidant properties. 100 In a model of myocardial IRI, aspirin use resulted in a marked decrease in nitro-oxidative stress and enhanced function of the endothelium. 101

#### P2Y _12_ Inhibitors

The P2Y _12_ receptor is present on platelets, as well as on leukocytes, vascular smooth muscle cells, or microglia. This broad distribution suggests that P2Y _12_ antagonists may also affect non-platelet P2Y _12_ receptors, potentially modulating vascular function and inflammatory responses. 102 Since inflammatory pathways are closely linked to oxidative stress, reducing inflammation may decrease ROS production, further limiting platelet activation. In addition to its role as a P2Y _12_ receptor antagonist, ticagrelor prevents adenosine uptake, enhancing adenosine-mediated effects. Adenosine, which protects the heart from IRI, inhibits platelet activation and suppresses ROS generation in reperfused tissues. 103 The mechanisms of action and effects of antiplatelet therapy on oxidative stress are summarized in Table 3 .

**Table 3:** Antiplatelet agents—mechanisms, oxidative stress, and therapeutic effects

Agent	Primary mechanism of action	Impact of oxidative stress on efficacy	Antioxidant/Anti-inflammatory effects	Clinical notes
**Aspirin**	Irreversibly inhibits COX-1 → blocks TXA _2_ synthesis	Bypasses COX-1 via isoprostanes 14 95 146 ; aspirin resistance in DM, smokers, inflammation 147 148	Inhibits prostaglandin synthesis and oxidative stress 99 ; induces HO-1 100 ; reduces nitro-oxidative stress 101	Foundational antiplatelet therapy; limited efficacy when used alone post-AMI 90
**Clopidogrel**	Prodrug; irreversibly inhibits P2Y _12_ receptor via active metabolite (CYP activation required)	Impaired CYP activation reduces efficacy 96 ; HTPR more common in high ROS states 97 98	Reduces lipid peroxidation; increases CT and total antioxidant capacity 149 ; mitigates IRI 150	Commonly used; genetic variability in response; slower onset
**Prasugrel**	Prodrug; irreversibly inhibits P2Y _12_ receptor (faster, more potent than clopidogrel)	Affected by CYP metabolism but less variability compared with clopidogrel	Improves endothelial NO bioavailability 151 ; reduces inflammation via neutrophil inhibition 151	Faster and more potent than clopidogrel; prodrug (CYP dependent)
**Ticagrelor**	Direct, reversible P2Y _12_ receptor inhibitor; also inhibits adenosine reuptake	Unaffected by CYP; more consistent inhibition under oxidative stress	Upregulates SOD, 152 reduces ROS; inhibits apoptosis via AKT/Nrf2/HO-1 and adenosine 153 154	Rapid onset; effective in oxidative stress; adenosine-mediated cardioprotection 103
**Cangrelor**	Intravenous, direct reversible P2Y _12_ receptor inhibitor with rapid onset/offset	Likely less affected due to direct action (limited clinical ROS-specific data)	Unknown—limited data on ROS-specific effects	Used in acute PCI; rapid-on/off kinetics ideal for procedural use

Abbreviations: AMI, acute myocardial infarction; COX, cyclooxygenase; CT, catalase; CYP, cytochrome; DM, diabetes mellitus; HO-1, heme oxygenase-1; HTPR, high on-treatment platelet reactivity; IRI, ischemia-reperfusion injury; NO, nitric oxide; PCI, percutaneous coronary intervention; ROS, reactive oxygen species; SOD, superoxide dismutase; TXA _2_ , thromboxane A _2_ .

## Impact of Antiplatelet Agents on Platelet miRNAs

### Aspirin

Antiplatelet therapy modifies the expression of miRNAs associated with oxidative stress and platelet function by regulating platelet activation and related intracellular signaling pathways. Plasma levels of several platelet miRNAs, including miRNA-223, miRNA-191, miRNA-126, miRNA-197, and miRNA-150, decrease upon platelet inhibition with aspirin. 78 104

### P2Y _12_ Inhibitors

In mice, miRNA-126 inhibition reduced TXA _2_ -dependent platelet aggregation and affected P2Y _12_ expression. 6 In patients, miRNA levels, including miRNA-126, miRNA-223, miRNA -21 and miRNA-150, correlated with the P2Y _12_ inhibitor used, with prasugrel and ticagrelor generally showing stronger suppression than clopidogrel. 105 106 In contrast, Jӓger et al reported that ticagrelor was associated with higher levels of miRNA-150, miRNA-21, and miRNA-126 compared with clopidogrel and prasugrel, while drug cessation did not alter miRNA expression despite increased ADP-mediated platelet activation. 107 Dose-escalation studies confirmed that most platelet miRNA levels decline as platelet inhibition intensifies. 5 Some, such as miRNA-96, increase with stronger platelet inhibition. 106 Prasugrel, but not clopidogrel, reduced miRNA-223, miRNA-24, miRNA-191, and miRNA-197 expression in diabetic patients compared with ASA, suggesting prasugrel may provide superior antiplatelet efficacy, particularly in patients with diabetes. 104 Table 2 shows selected miRNAs and their changes in response to antiplatelet therapy.

## Clinical Use

### Monitoring of Therapy

European Society of Cardiology treatment guidelines advocate the use of P2Y _12_ inhibitors such as ticagrelor or prasugrel instead of clopidogrel because of their greater potency, quicker onset of action, and reduced interindividual variability, though they carry an increased bleeding risk. 93 108 Despite this, clopidogrel is still commonly used, though 4 to 30% of patients exhibit inadequate responses, increasing cardiovascular risk. 109 Monitoring DAPT strategies can help identify such patients and guide treatment adjustments to balance ischemic and bleeding risks.

Several methods assess platelet activity, but no single test is ideal for routine use. 110 111 The vasodilator-stimulated phosphoprotein (VASP) phosphorylation assay is highly specific for P2Y _12_ inhibition, with minimal interference from aspirin or GPIIb/IIIa antagonists and is more precise than traditional light transmission aggregometry (LTA). 112 Other methods, like VerifyNow and multiplate, show modest correlation, with the best comparability between LTA and VASP. 113 114 Biomarkers like platelet–leukocyte aggregates, CD40L, and sP-selectin may also assess platelet activation. 115 116 Flow cytometry using monoclonal antibodies for platelet activation or adhesion markers like CD41 (GP IIb/IIIa), CD42b (GP Ib), CD40L, or CD63 can assess platelet activation. 117 However, methods like LTA are time-consuming and technically challenging, while sP-selectin may have limited detectability. Immunological methods can also be costly.

Monitoring oxidative stress markers and miRNAs involved in thrombotic processes, such as platelet activation and coagulation, can provide an alternative approach to assessing thrombotic risk and evaluating antithrombotic therapy efficacy. Increased or decreased levels of miRNAs like miRNA-126 and miRNA-223 correlate with thrombotic risk, while their change after antithrombotic treatment could indicate therapeutic success ( Table 2 ).

### Diagnostic Markers and Outcome Predictors

Studies have shown that miRNA-223, miRNA-19b, and miRNA-483–5p provide higher diagnostic accuracy than troponin for early AMI diagnosis. 118 Additionally, miRNA-24–1 and miRNA-545 were identified as potential diagnostic biomarkers for AMI. 119 miRNA-223–3p, miRNA-197, and miRNA-126–3p show potential as independent indicators of thrombosis and ischemic complications post-AMI. 89 120 121 When combined with the Framingham Risk Score, these miRNAs enhance risk stratification. 86 These findings suggest that combining these markers with those currently in use may enhance diagnostic and therapeutic accuracy, especially in high-risk populations. Additionally, using miRNA primers for quantitative real-time PCR is less time- and cost-intensive than developing specific antibodies or ELISAs. 122

There are limitations to using miRNAs as biomarkers. Most miRNAs implicated in CVDs are not platelet-specific and are found in other cells, such as endothelium, leukocytes, cardiomyocytes, and hematopoietic stem cells. 123 Conditions like hypertension, hyperlipidemia, smoking, and diabetes can affect miRNA levels, including miRNA-126, miRNA-146a, miRNA-223, and miRNA-214, as well as oxidative stress markers. In diabetes, downregulation of miRNAs critical for platelet activation can lead to upregulation of P2Y _12_ receptors, reducing the efficacy of P2Y _12_ -targeting antiplatelet drugs. 124 Oxidative modifications can also make platelets more resistant to endogenous antiplatelet mechanisms, increasing thrombotic risk. The complexity and diverse sources of miRNAs can complicate their use as platelet activity markers, but this may also offer valuable insights into underlying processes.

## Conclusion

Oxidative stress significantly alters platelet function after AMI by promoting platelet activation and aggregation, while impairing endothelial function and reducing NO bioavailability. These changes highlight the importance of targeting oxidative stress in managing thrombotic complications post-AMI.

Antiplatelet therapy plays a key role in treating AMI and its complications. Aspirin and P2Y _12_ receptor inhibitors not only exhibit antiplatelet activity but also help mitigate oxidative stress, inflammation, and myocardial injury. Integrating oxidative stress markers and miRNA-based biomarkers holds promise for monitoring antithrombotic efficacy, assessing thrombotic risk, and evaluating treatment response.

miRNAs offer potential as therapeutic targets for modulating thrombotic processes. Manipulating miRNA expression or activity could enhance existing therapies, improve outcomes, reduce adverse effects, and enable personalized treatment strategies.
