# Supplementary material for: The Role of Oxidative Stress and MicroRNAs in Platelet Activation and the Efficacy of Antiplatelet Therapy in Acute Myocardial Infarction
Source: Thromb Haemost. 2025 Nov 27;126(11):1084–95. doi: 10.1055/a-2741-4754 (PMC13722529; doi:10.1055/a-2741-4754)
Supplement: Supplementary file 1 [file 10-1055-a-2741-4754_27579043.pdf]

Table S1. Antioxidant systems regulating platelet redox balance and function

Antioxidant System	Location/Source	Mechanism of action	Effect on platelets
**Endogenous antioxidant systems**			
**Superoxide dismutase**	Platelets (SOD 1, SOD 2), plasma	Dismutates superoxide into oxygen and hydrogen peroxide; enhances NO bioavailability [1]	Inhibits activation; supports thrombus prevention [2,3]; reduced activity in AMI [4]
**Catalase**	Platelet cytosol, plasma	Decomposes hydrogen peroxide into water and oxygen; prevents hydroxyl radical formation	Protects against ROS damage; inhibits platelet aggregation [5]; reduced after AMI reperfusion
**Glutathione peroxidase**	Platelets, plasma (GPx3)	Reduces hydrogen peroxide and lipid hydroperoxides using GSH; forms GSSG	Enhances the inhibition of platelet function [6]; deficiency increases lipid peroxidation and thrombosis risk [7]; increased in AMI [8]
**Glutathione**	Platelets, plasma (GSH/GSSG couple)	Serves as cofactor for GPx; maintains redox balance by converting ROS to less reactive forms	Depletion impairs GPx function and redox homeostasis [6]
**Heme oxygenase-1**	Low expression in platelets, plasma	Degrades heme to biliverdin, iron, and CO; CO inhibits platelet aggregation	Reduces platelet activation under oxidative stress [9]
**Thioredoxin**	Platelets, plasma	Reduces disulfide bonds; modulates NOX-2 and redox-sensitive platelet receptors (TXA_2 and the $P2Y_{12}$ receptors) [10]	Correlates with increased aggregability in AMI; protects against apoptosis [11]
**Exogenous antioxidant systems**			
**Endothelial-derived NO**	Endothelium	Produces NO to inhibit platelet activation and thrombus formation	Inhibits platelet aggregation; promotes vasodilation [12]

**Vascular antioxidants**	Plasma	Enzymatic; neutralize ROS; protect membrane proteins/lipids; modulate signaling pathways	Reduce platelet reactivity; lowers thrombotic risk [13]
**Dietary antioxidants** (vitamins, polyphenols, lignans, stilbenes etc.)	Plasma, diet	Non-enzymatic; neutralize ROS; protect membrane proteins/lipids; modulate signaling pathways	Reduce platelet reactivity; lowers thrombotic risk [14]

Abbreviations: **SOD-** superoxide dismutase; **NO-** nitric oxide; **AMI-** acute myocardial infarction; **ROS-** reactive oxygen species; **GPx-** glutathione peroxidase; **GSH** -glutathione; **GSSG-** oxidized glutathione; **CO-** carbon monoxide; **NOX-** NADPH oxidase; **TXA_2-** thromboxane A_2

References

1. Meng YY, Trachtenburg J, Ryan US, Abendschein DR. Potentiation of endogenous nitric oxide with superoxide dismutase inhibits platelet-mediated thrombosis in injured and stenotic arteries. *J Am Coll Cardiol*. 1995;25(1):269-275. doi:10.1016/0735-1097(94)00349-u

2. Leo R, Praticò D, Iuliano L, et al. Platelet activation by superoxide anion and hydroxyl radicals intrinsically generated by platelets that had undergone anoxia and then reoxygenated. *Circulation*. 1997;95(4):885-891. doi:10.1161/01.cir.95.4.885

3. Krötz F, Sohn HY, Gloe T, et al. NAD(P)H oxidase-dependent platelet superoxide anion release increases platelet recruitment. *Blood*. 2002;100(3):917-924. doi:10.1182/blood.v100.3.917

4. Pandey NR, Kaur G, Chandra M, Sanwal GG, Misra MK. Enzymatic oxidant and antioxidants of human blood platelets in unstable angina and myocardial infarction. *Int J Cardiol*. 2000;76(1):33-38. doi:10.1016/s0167-5273(00)00344-2

5. Del Principe D, Menichelli A, De Matteis W, Di Corpo ML, Di Giulio S, Finazzi-Agro A. Hydrogen peroxide has a role in the aggregation of human platelets. *FEBS Lett*. 1985;185(1):142-146. doi:10.1016/0014-5793(85)80758-4

6. Freedman JE, Frei B, Welch GN, Loscalzo J. Glutathione peroxidase potentiates the inhibition of platelet function by S-nitrosothiols. *J Clin Invest*. 1995;96(1):394-400. doi:10.1172/JCI118047

7. Jin RC, Mahoney CE, Coleman Anderson L, et al. Glutathione peroxidase-3 deficiency promotes platelet-dependent thrombosis in vivo. *Circulation*. 2011;123(18):1963-1973. doi:10.1161/CIRCULATIONAHA.110.000034

8. Holley A, Pitman J, Miller J, Harding S, Larsen P. Glutathione peroxidase activity and expression levels are significantly increased in acute coronary syndromes. *J Investig Med Off Publ Am Fed Clin Res*. 2017;65(5):919-925. doi:10.1136/jim-2016-000361

9. Peng L, Mundada L, Stomel JM, et al. Induction of heme oxygenase-1 expression inhibits platelet-dependent thrombosis. *Antioxid Redox Signal*. 2004;6(4):729-735. doi:10.1089/1523086041361677

10. Essex DW. The role of thiols and disulfides in platelet function. *Antioxid Redox Signal*. 2004;6(4):736-746. doi:10.1089/1523086041361622

11. Miyamoto S, Sakamoto T, Soejima H, et al. Plasma thioredoxin levels and platelet aggregability in patients with acute myocardial infarction. *Am Heart J*. 2003;146(3):465-471. doi:10.1016/S0002-8703(03)00311-9

12. Naseem KM, Riba R. Unresolved roles of platelet nitric oxide synthase. *J Thromb Haemost JTH*. 2008;6(1):10-19. doi:10.1111/j.1538-7836.2007.02802.x

13. Scioli MG, Storti G, D'Amico F, et al. Oxidative Stress and New Pathogenetic Mechanisms in Endothelial Dysfunction: Potential Diagnostic Biomarkers and Therapeutic Targets. *J Clin Med*. 2020;9(6):1995. doi:10.3390/jcm9061995

14. Giurranna E, Nencini F, Bettiol A, et al. Dietary Antioxidants and Natural Compounds in Preventing Thrombosis and Cardiovascular Disease. *Int J Mol Sci*. 2024;25(21):11457. doi:10.3390/ijms252111457
